# A Good Bracket

**Published:** 1882-02

**Authors:** 


### A GOOD BRACKET.

What bracket do I use? Well, I have used almost every style of bracket that has been presented to the profession; as improvements were made new ones were substituted, and usually each was better than the preceding; the good points increasing, and the defects becoming less, till now in the Spencer & Crocker bracket recently improved the defects have all disappeared. It is about all that can be desired in such a fixture. It has every movement necessary; it works easily, is steady in any position in which it is placed; it is strong and cannot get out of repair by any legitimate usage. It, or something equally good, ought to supersede the multitude of defective, rickety and annoying things, that the profession has been compelled to use. The profession owes a debt of gratitude, at least to Mr. Crocker, for the valuable improvements he has made in the dental bracket. In behalf of the profession, I feel impelled to say thus much of this appliance, after having made a thorough test of it.

